# The wing of the ToxR winged helix-turn-helix domain is required for DNA binding and activation of *toxT* and *ompU*

**DOI:** 10.1371/journal.pone.0221936

**Published:** 2019-09-09

**Authors:** Sarah J. Morgan, Emily L. French, Sarah C. Plecha, Eric S. Krukonis

**Affiliations:** 1 Department of Microbiology and Immunology, University of Michigan, Ann Arbor, MI, United States of America; 2 Division of Integrated Biomedical Sciences, University of Detroit Mercy School of Dentistry, Detroit, MI, United States of America; 3 Department of Biochemistry, Microbiology and Immunology, Wayne State University, Detroit, MI, United States of America; University of Manchester, UNITED KINGDOM

## Abstract

ToxR and TcpP, two winged helix-turn-helix (w-HTH) family transcription factors, co-activate expression of the *toxT* promoter in *Vibrio cholerae*. ToxT then directly regulates a number of genes required for virulence. In addition to co-activation of *toxT*, ToxR can directly activate the *ompU* promoter and repress the *ompT* promoter. Based on a previous study suggesting that certain wing residues of ToxR are preferentially involved in *toxT* co-activation compared to direct *ompU* activation, we employed alanine-scanning mutagenesis to determine which residues in the wing of ToxR are required for activation of each promoter. All of the ToxR wing residues tested that were critical for transcriptional activation of *toxT* and/or *ompU* were also critical for DNA binding. While some ToxR wing mutants had reduced interaction with TcpP, that reduced interaction did not correlate with a specific defect in *toxT* activation. Rather, such mutants also affected *ompU* activation and DNA binding. Based on these findings we conclude that the primary role of the wing of ToxR is to bind DNA, along with the DNA recognition helix of ToxR, and this function is required both for direct activation of *ompU* and co-activation of *toxT*.

## Introduction

ToxR and TcpP are transmembrane transcription factors that coordinately activate transcription of *toxT*, the gene encoding the master virulence regulator in *V*. *cholerae*. TcpP is the direct activator of *toxT*, binding to the *toxT* promoter from -53 to -38, just upstream of the -35 element [1]. Although, TcpP is able to activate intermediate levels of *toxT* expression when overexpressed in the absence of ToxR [2, 3], ToxR is required for TcpP-mediated expression of *toxT* at endogenous expression levels. ToxR binds the *toxT* promoter from -96 to -83 [4], approximately three helical turns upstream of the TcpP binding site, enhancing TcpP-mediated activation of the *toxT* promoter. In addition to acting as a co-activator of *toxT*, ToxR is also able to directly activate the *ompU* promoter and repress the *ompT* promoter [4–6].

**Fig 1:**
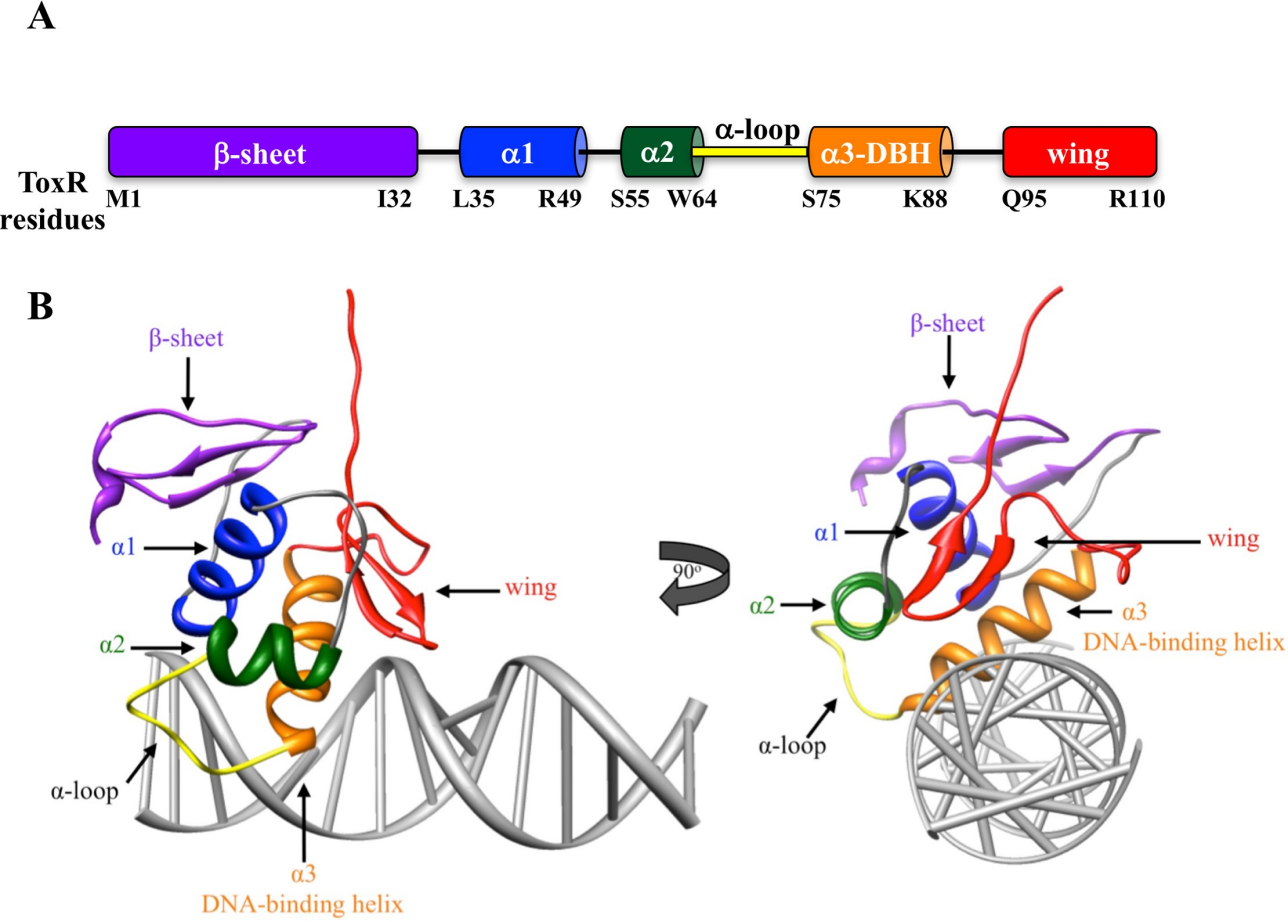
Domain arrangement of ToxR and modeled structure of ToxR on DNA highlighting those domains. A) Based on homology to other w-HTH proteins [7] the residues defining each domain of ToxR are highlighted. B) A modeled structure of ToxR bound to DNA was generated with the I-TASSER modeling program (http://zhanglab.ccmb.med.umich.edu/I-TASSER/) and the crystal structure of other w-HTH family members [26]. Binding of ToxR to DNA was modeled using the NMR structure of PhoB bound to DNA [25]. Domains were highlighted with the same color scheme used in part A.

ToxR and TcpP both have cytoplasmic domains homologous to the w-HTH (winged helix-turn-helix) family of transcription factors [7]. Most w-HTH proteins have an N-terminal regulatory domain and a C-terminal w-HTH domain. However, both ToxR and TcpP have an N-terminal w-HTH domain, which is linked to a C-terminal periplasmic domain through a single-pass transmembrane domain. The periplasmic domain of ToxR is involved in, but not required for dimerization [8–13]. The periplasmic domain of TcpP regulates stability and is proteolytically degraded under non-inducing conditions [14–17]. Likewise, the periplasmic domain of ToxR is the target of proteolysis under conditions of alkaline pH and nutrient limitation [18]. The functions and stability of ToxR and TcpP periplasmic domains are enhanced by the periplasmic proteins ToxS and TcpH, respectively [8, 14–17, 19, 20]. The w-HTH domains of ToxR and TcpP bind to the *toxT* promoter and activate transcription. The w-HTH domain consists of an N-terminal β-sheet, 3 α-helixes including the DNA-binding helix (α3) that is inserted into the major groove of the DNA, and a C-terminal wing (Fig 1). The N-terminal β-sheet can be involved in protein-protein interaction as well as stabilizing the hydrophobic core [7, 21–23]. The first two α-helixes form part of the hydrophobic core as well as interact with the DNA backbone helping to stabilize protein-DNA interactions [21, 24, 25]. Between the second α-helix (α2) and the DNA-binding helix (α3) is the α-loop. The α-loop of w-HTH proteins is hypothesized to interact with RNA polymerase (RNAP, [7]). The α-loop of ToxR is critical for direct activation of the *ompU* promoter while less essential for co-activation of the *toxT* promoter, as would be predicted for a domain that interacts with RNAP [26]. The wing of w-HTH proteins consists of a β-strand hairpin, which inserts into the minor grove of the DNA, thereby enhancing binding to the promoter [21, 25, 27]. The wing of w-HTH proteins is often also involved in dimerization [21, 28, 29].

Dimerization is critical for activation of w-HTH transcription factors and can be mediated by interactions between w-HTH domains, the N-terminal regulatory domain (of w-HTH proteins containing this domain) and the promoter architecture [21, 23, 28, 30, 31]. w-HTH proteins have been found to dimerize in three different orientations. Both PhoB and OmpR have been shown to dimerize in a head-to-tail orientation on the promoter. In this orientation, the wing of the upstream w-HTH protein interacts with the β-sheet of the downstream w-HTH [21, 28]. Alternatively, *Mycobacterium tuberculosis* PhoP has been shown by crosslinking to dimerize in a head-to-head orientation with the two β-sheets interacting [30]. OmpR can also be found in head-to head orientations [23], although formation of OmpR dimers in head-to-head or head-to-tail orientation may be primarily dependant on the orientation of the OmpR binding site [31]. Finally, HSF in *Kluyveromyces lactis* interacts in a tail-to-tail orientation in which the two wings interact [29]. In this system the wings are only involved in protein-protein interaction and do not appear to interact with the DNA [29]. Both ToxR and TcpP form homodimers [8–13, 32] as well as ToxR-TcpP heterodimers [2, 26, 33]. However, the orientation and interface(s) of these dimers has not been elucidated.

When ToxR is bound to the *toxT* promoter, a hypersensitivity site is created at the TcpP binding site [34] indicating an alteration of the structure of the *toxT* promoter upon ToxR binding. This hypersensitivity site indicates that upon ToxR binding to the *toxT* promoter, the TcpP-binding site may become more accessible, allowing for enhanced TcpP binding to the promoter. Although, it should be noted when membranes containing both ToxR and TcpP were used for DNAse I footprinting studies, only the ToxR-protected region was observed [34]. This suggests ToxR may bind DNA and tether TcpP in place for RNAP recruitment/stimulation [2, 26].

Additionally, the *toxT* promoter is repressed by H-NS, and ToxR is critical for *toxT* activation in the presence of H-NS, as it can increase expression of *toxT* over 10-fold, whereas in the absence of H-NS, ToxR only increases expression of *toxT* two to three-fold [35]. Together, this indicates that one of the primary roles of ToxR may be to relieve H-NS repression, but that ToxR also plays an additional roll(s) in activation beyond relieving H-NS repression. ToxR also likely influences the localization of the *toxT* promoter by recruiting it to the membrane thereby enhancing access of TcpP to the *toxT* promoter [36]. Although a soluble form of the cytoplasmic domain of ToxR is able to activate transcription of *ompU*, membrane localization is required for ToxR co-activation of *toxT* [37].

Another possible role for ToxR in co-activation of TcpP is recruitment of TcpP to the *toxT* promoter through ToxR-TcpP interaction. ToxR-TcpP interaction can be observed by DSP crosslinking in *V*. *cholerae* membranes [2, 26] and a membrane-localized *E*. *coli* bacterial two-hybrid (BACTH) system [26]. ToxR-TcpP interaction likely occurs via the wing of TcpP, since mutations in the wing of TcpP can disrupt ToxR-TcpP interaction resulting in a defect in transcriptional activation [2]. The wing of TcpP plays a dual role, since residues in the wing of TcpP also are required for binding to the *toxT* promoter [2, 34]. The wing of ToxR may also be involved in ToxR-TcpP interaction, as ToxR-P101L was preferentially defective for *toxT* activation, relative to *ompU* activation, and was defective in interaction with TcpP by DSP crosslinking [26]. It is possible ToxR-TcpP interaction is maintained upon binding the *toxT* promoter, allowing ToxR to stabilize TcpP binding to the promoter, thereby enhancing transcriptional activation. Alternatively, ToxR-TcpP interaction could be disrupted upon ToxR binding to DNA, allowing each protein to then bind the promoter independently. Finally, it is possible that ToxR/TcpP interaction is maintained upon DNA binding by ToxR. In this scenario, TcpP would be tethered in place such that it could productively interact with RNA polymerase without binding DNA. This model is supported by the observation that a poor DNA-binding mutant of TcpP, TcpP-H93L, can be rescued for *toxT* activation when co-expressed with ToxR [2]. This model is also supported by the observation that TcpP-mediated footprinting is lost in the presence of ToxR co-expression [34].

The role of the ToxR-wing in co-activation of *toxT* could be due to DNA binding and/or ToxR-TcpP interaction. DNA binding by the wing of ToxR is critical for activation of both *toxT* and *ompU* since mutations in the wing of ToxR (T99K or G104S) that inhibit DNA binding inhibit transcriptional activation of both promoters [26]. However, several mutants in the wing (P101L, and R103G) as well as mutants in the loop leading from the DNA-binding helix to the wing (D89E, K92E, S93P) are preferentially defective for transcription of *toxT*, compared to *ompU* [26]. This could be due to differences in binding to the two promoters, ToxR-TcpP interaction, or other as yet unknown factors. The goal of this study was to determine the role of each residue in the wing of ToxR in transcriptional activation of both *toxT* and *ompU*. The requirement of ToxR wing residues for ToxR-TcpP interaction and DNA binding was also assessed to determine what role the wing of ToxR plays in co-activation of *toxT*.

## Results

### The wing of ToxR is required for transcriptional activation of both *toxT* and *ompU*

**Fig 2:**
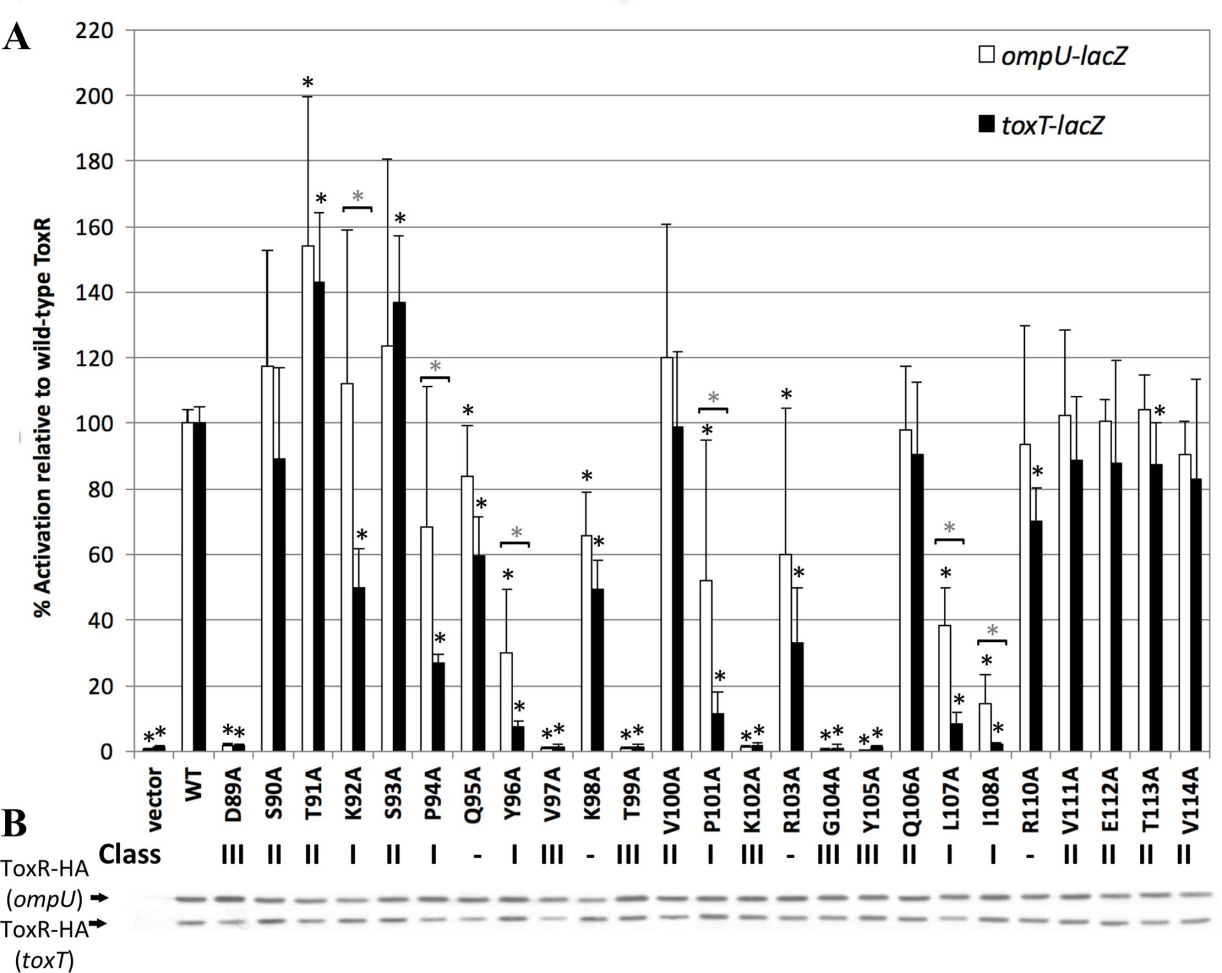
Requirement of each residue in the ToxR wing on *toxT* and *ompU* activation. A) ToxR-HA wing mutants were expressed from pBluescriptSK- (pSK) in the presence of pREP4 to regulate expression. The level of activation both the *toxT-lacZ* and *ompU-lacZ* chromosomal reporters for each mutant was measured by β-galactosidase assay and normalized to wild-type ToxR. * p<0.001923 using the Students’ T-test and a cut-off for significance at 0.05/26 according to the Bonferroni correction for multiple comparisons. * above a [indicates ToxR mutants that are at least 2-fold more defective for *toxT* activation than *ompU* activation. * p<0.05 using the Students’ T-test. B) Stability of ToxR-HA was monitored by Western blot using a monoclonal anti-HA. All strains were tested at least six times on at least two different days. Where appropriate, the mutant class is listed below each substitution. ToxR-Q95A, ToxR-K98A, ToxR-R103A, and ToxR-R110A did not fit into any of the mutant class designations.

To determine the role of each wing residue in ToxR co-activation of *toxT* and direct activation of *ompU*, we mutated each wing residue to alanine, including residues in the loop between the DNA-binding helix (α3) and the wing (Fig 1). The range of potential wing residues from D89-V114 was based on homology of ToxR to molecules with known structures such as OmpR and PhoB {Krukonis, 2000 #5068}. Alanine scanning was used because residues in this region of ToxR can have different phenotypes depending on the amino acid to which they are mutated [26]. Each ToxR-wing mutant was expressed from a plasmid and tested for transcriptional activation of chromosomal *ompU-lacZ* (strain EK410) and *toxT-lacZ* (strain EK1072) (Fig 2) [26, 37]. Since each ToxR derivative was tagged at the C-terminus (periplasmic domain) with an HA tag, expression of each ToxR mutant was assessed in both the *ompU-lacZ* and *toxT-lacZ* reporter strains with an anti-HA antibody (Fig 2B).

**Fig 3:**
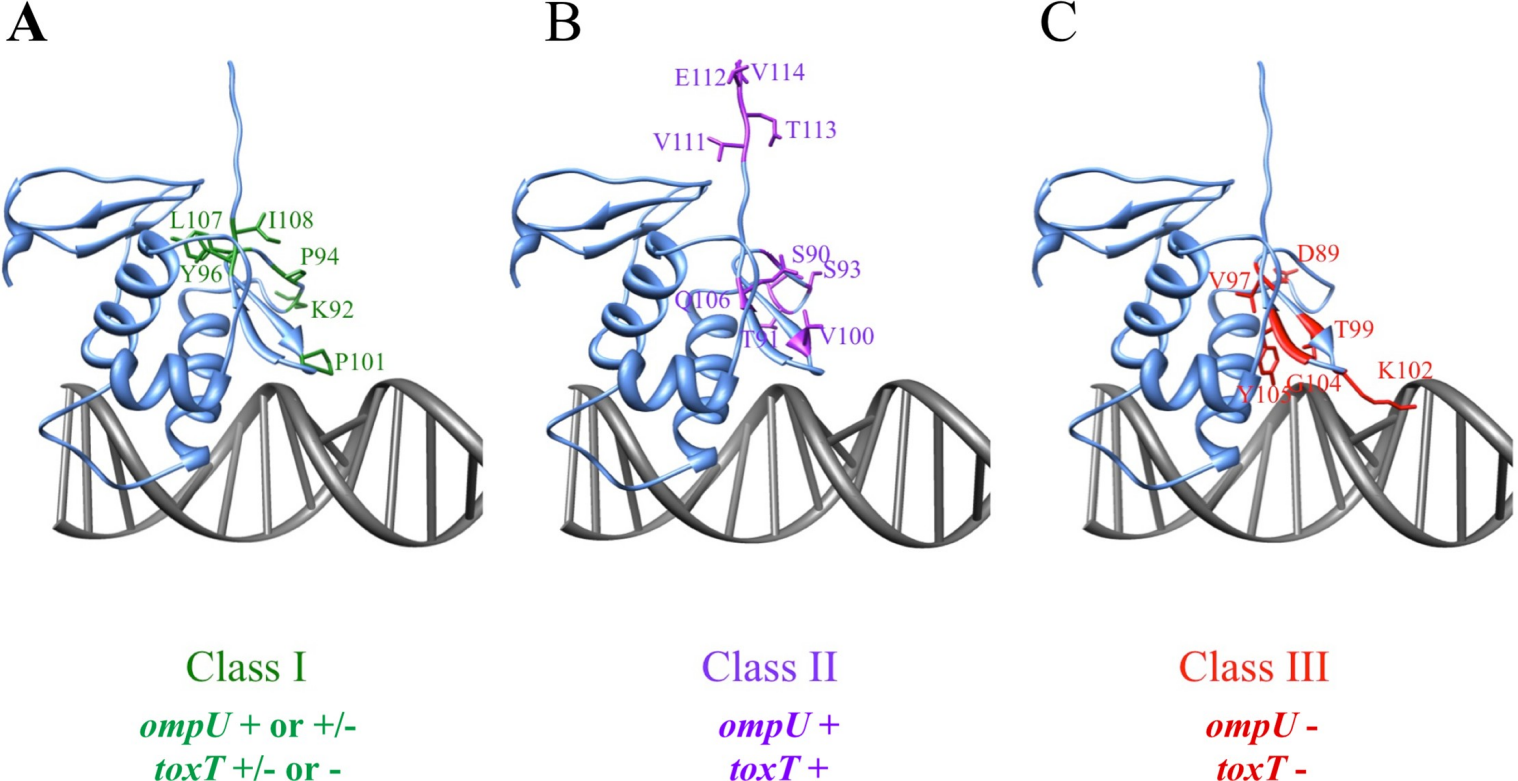
Assignment of three classes of ToxR wing mutants. ToxR w-HTH domain was previously modeled using I-TASSER (http://zhanglab.ccmb.med.umich.edu/I-TASSER/)[26]. This previously-generated model with then modeled onto DNA using Chimera and the NMR structure of the ToxR homolog PhoB bound to DNA {Yamane, 2008 #5306}. Mutants were divided into classes based on phenotype. A) Class I mutants (green) are greater than 2-fold defective for *toxT* activation relative to *ompU* activation when expressed from a plasmid (Fig 2). B) Class II mutants (purple) are not significantly defective for activation of either promoter. C) Class III mutants (red) are required for activation of both *toxT* and *ompU* promoters and DNA binding.

Of the 25 residues tested, six were preferentially required for *toxT* co-activation as compared to *ompU* activation (K92, P94, Y96, P101, L107, and I108, Fig 2). Residues preferentially required for *toxT* co-activation were defined by mutation of that residue resulting in a 2-fold decrease in *toxT* co-activation relative to *ompU* activation and designated Class I mutants (Fig 3 green). Three of the six residues (Y96, L107, and I108) were required for both *toxT* co-activation and *ompU* activation as mutation of these residues resulted in less than 50% activation of *ompU* (Fig 2). These three residues are particularly critical for co-activation of *toxT*, since mutation of any of these residues decreased *toxT* transcriptional activation to 10% or less of wild-type. Based on *in silico* modeling, these residues are clustered at the hinge of the wing adjacent to the β-strands (Fig 3A). The two prolines present in the wing, P94 and P101, are partially required for *ompU* activation (70% and 50% activity respectively, when mutated), but critical for *toxT* co-activation (26% and 11% activity respectively, when mutated). K92 is not required for *ompU* activation (maintained ~100% activity when mutated), but is involved in *toxT* co-activation (50% activity), similar to a previously described ToxR-K92E mutant [26]. None of the wing residues were preferentially required for *ompU* activation relative to *toxT* activation (Fig 2).

Several residue side chains in the wing region of ToxR are not required for transcriptional activation of either promoter (S90, T91, S93, V100, Q106, V111, E112, T113, and V114, Fig 2) and were designated Class II mutants (Fig 3B, purple). When these residues were mutated to alanine >80% of transcriptional activation of both promoters was maintained (Fig 2). The majority of these residues are either in the loop between the DNA-binding helix (α3) and the wing or after the β-hairpin of the wing (Fig 3). The only amino acid side-chain in the β-turn of the wing (residues V97-G104) not required for transcriptional activation of at least one promoter was V100, indicating that the β-hairpin is critical for transcriptional activation. This is expected since many w-HTH proteins insert the β-hairpin of the wing into the minor groove of promoter DNA [21, 25, 27].

As was expected, since the wing of ToxR is involved in DNA binding [26], several wing residues of ToxR are required for transcriptional activation of both *ompU* and *toxT* (D89, V97, T99, K102, G104, Y105, Fig 2). Mutation of these residues, designated Class III (Fig 3C), resulted in less than 5% activation of either *ompU* or *toxT* loci (Fig 2). This results in *toxT* activation as low as the empty vector control. The majority of these residues are predicted to be involved in DNA binding, either directly or indirectly, and mutations in T99 and G104 have been previously shown to disrupt DNA binding to both promoters [26]. All of the residues required for any detectable transcriptional activation of both promoters are found in the β-hairpin of the wing except D89, which is positioned near the end of the DNA-binding helix (Fig 3C).

### Analysis of selected ToxR mutants expressed from the chromosome

**Fig 4:**
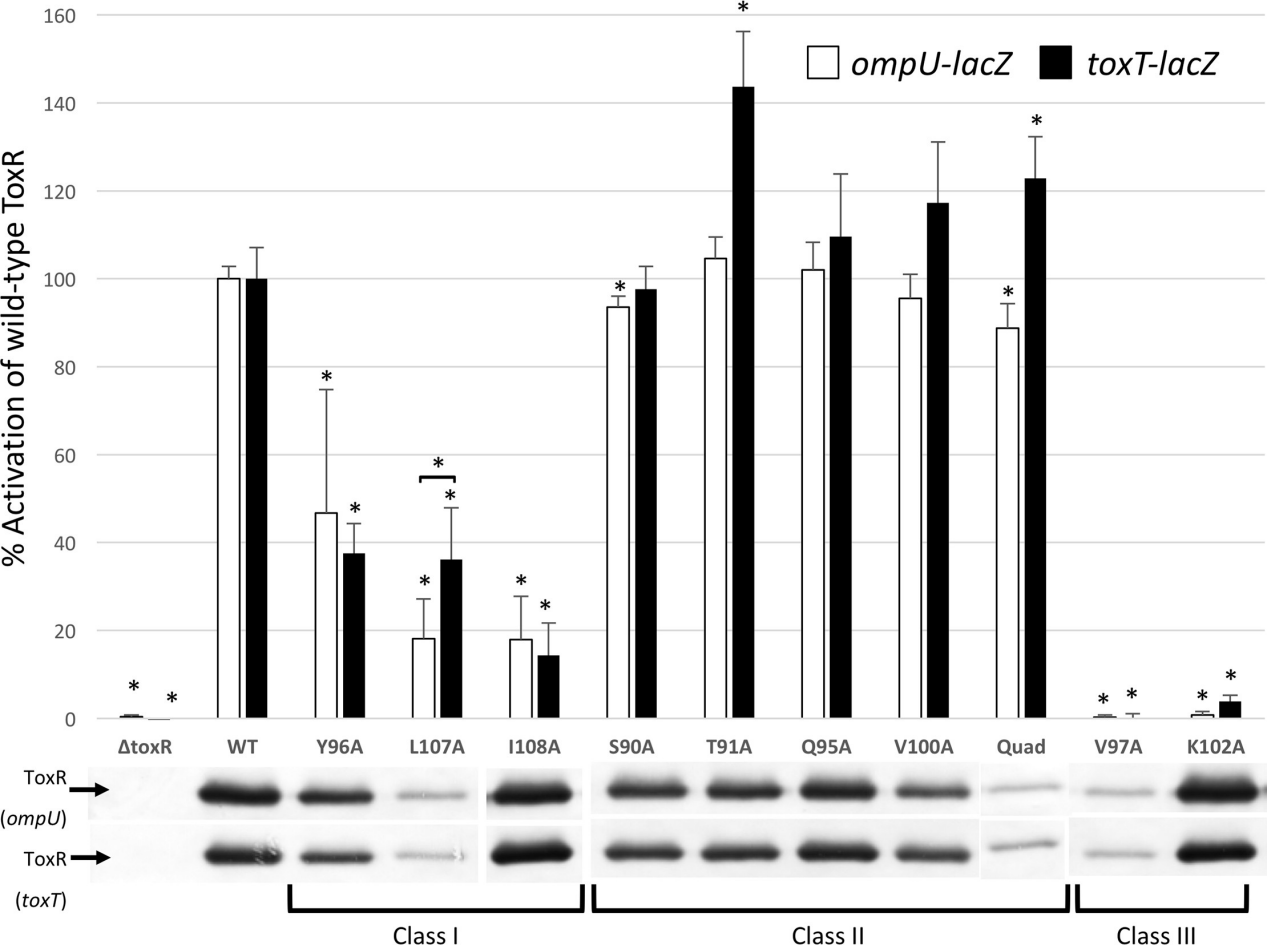
ToxR wing mutants expressed from the *toxR* chromosomal locus are not preferentially defective for *toxT* activation. A) Selected ToxR wing mutants representing each class of mutant were placed on the chromosome and assayed for transcriptional activation of *toxT-lacZ* and *ompU-lacZ* by β-galactosidase assay. Class I mutants were at least 2-fold more defective for *toxT* activation than *ompU* activation when expressed from a plasmid, but equally defective for activation of both promoters when endogenously expressed. Class II mutants are not defective for activation of either promoter, and maintained ≥90% activity even when mutated. Class III mutants are defective for activation of both promoters. * p<0.0045 using the Students’ T-test and a cut-off for significance at 0.05/11 according to the Bonferroni correction for multiple comparisons. B) The stability of each ToxR wing mutant was monitored by Western blot using anti-ToxR antibodies.

Since overexpression can result in altered phenotypes, we analyzed the requirement for certain ToxR wing residues for transcriptional activation using chromosomally expressed ToxR wing mutants. Thus, we measured activation of chromosomal *toxT-lacZ* and *ompU-lacZ* reporter from members of each class of ToxR mutant expressed from the native *toxR* locus (Fig 4). Three residues preferentially required for *toxT* co-activation under overexpression conditions, Y96, L107, and I108, were no longer preferentially required for *toxT* co-activation when expressed from the chromosome. L107 was even slightly more critical for *ompU* activation than *toxT* co-activation when endogenously expressed (p<0.005), although ToxR-L107A was unstable when expressed from the chromosome in the absence of the C-terminal HA tag. Q95 contributed to, but was not required for, *toxT* activation when expressed from a plasmid (ToxR-Q95A has 59% of wild-type activation), was able to activate both *toxT* and *ompU* promoters at wild-type levels when chromosomally expressed (Figs 2 and 4).

All four class II mutants tested tolerated mutation to alanine and maintained transcriptional activation of both *toxT* and *ompU* promoters, whether expressed from the native *toxR* locus or a plasmid. To reveal any subtle effects on transcription by the four chromosomal class II mutants we constructed a quadruple mutant, ToxR-S90A/T91A/Q95A/V100A - designated ToxR-Quad, however this mutant was still fully able to activate both promoters (Fig 4). As was found with plasmid-based expression, both of the class III mutants tested (ToxR-V97A and ToxR-K102A) were still required for transcriptional activation of *toxT* and *ompU* when endogenously expressed.

These results illustrate that when one finds subtle phenotypes affecting activation of different promoters, assessing the affects with endogenous expression levels of the transcription activator can be informative. For these studies with un-tagged ToxR mutants expressed from the chromosome, the stability of three mutants appeared compromised, ToxR-V97A, ToxR-L107A and the ToxR-Quad mutant. However, since we used an anti-ToxR antibody to detect ToxR for this analysis, it remains possible that these mutations affected an epitope recognized by our antibody. V97 and L107 are predicted to be adjacent to one another at the hinge region of the ß-turn of the wing and may form a single epitope (Fig 3). This is why we used HA-tagged ToxR expressed from a plasmid for our initial studies.

### Several ToxR wing residues are required for DNA binding

**Fig 5:**
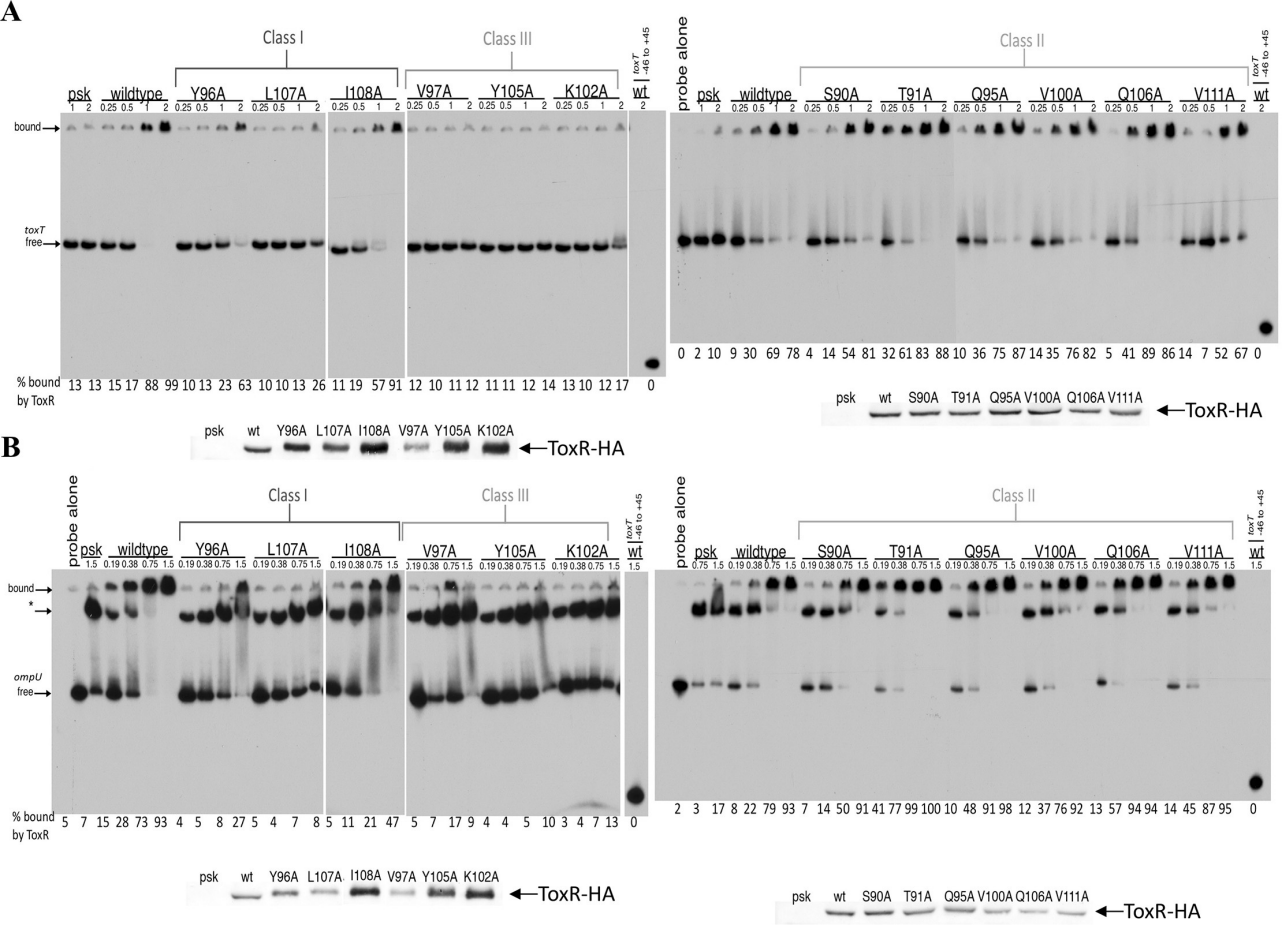
Several wing residues of ToxR are required for DNA binding. DNA binding by ToxR wing mutants was monitored using EMSA. *V*. *cholerae* membranes containing the ToxR wing mutants, but lacking TcpP, were bound to ^32^P end-labeled probes containing either the *toxT* promoter (A) or the *ompU* promoter (B). ToxR levels in each membrane preparation was monitored by anti-HA Western blot. Representative gels and quantification are shown from a minimum of two replicates. * indicates an intermediate ToxR-independent shifted *ompU* promoter that is also present with the pSK vector control extract [26].

Since the wing of w-HTH proteins is often involved in binding to the minor groove of the DNA, we determined which ToxR wing residues are required for DNA binding. We selected ToxR wing mutants from each class of ToxR wing residues and measured their DNA binding by EMSA (Electrophoretic Mobility Shift Assay) using plasmid-based expression of ToxR-HA derivatives. Each ToxR derivative was expressed in *V*. *cholerae* membranes and purified membranes were used for EMSAs as described previously {Krukonis, 2000 #5068}. Shifting of the *toxT* and *ompU* promoters by wild-type ToxR was detectable at 0.5 mg/ml and 0.19 mg/ml respectively (Fig 5A and 5B). All of the class III mutants tested (ToxR wing residues required for both *toxT* and *ompU* transcriptional activation) showed a dramatic decrease in binding capacity. This included ToxR-V97A, ToxR-Y105A, and ToxR-K102A that had no *toxT* binding capacity (Fig 5A and S1 Fig) and no shifting to the fully bound *ompU*/ToxR complex (Fig 5B and S1 Fig). As noted previously [26], even extracts from cells carrying the empty vector pSK-Bluescript had residual ToxR-independent *ompU* shifting to an intermediate position in the gel denoted with *.

The class I residues, which are involved in activation of both promoters, but preferentially affect *toxT* activation when expressed from a plasmid, were also required for DNA binding (Fig 5 and S1 Fig). High levels of membrane containing ToxR-Y96A or ToxR-I108A were required to shift the *toxT* and *ompU* promoters, indicating a dependence upon these residues for efficient DNA binding. Furthermore, these ToxR derivatives were more concentrated in their respective membrane preparations relative to wild-type ToxR, yet still required more membrane than wild-type ToxR to elicit similar gel shift activity (Fig 5, see anti-ToxR-HA Western blots). Mutation of ToxR-L107 resulted in an inability of ToxR-L107A to shift 50% of either probe even at the highest concentrations tested, indicating that this residue is a key residue for DNA binding (Fig 5). Thus, all of the ToxR wing residues tested for DNA binding that were involved in activation were also involved in promoter binding.

Of the class II mutants tested whose affected side-chains are not required for transcriptional activation of either promoter (ToxR-S90A, T91A, Q95A, V100A, Q106A, and V111A), none were defective for DNA binding either the *toxT* or *ompU* promoter (Fig 5 and S1 Fig). ToxR-T91A even had increased DNA binding efficiency to both the *ompU* and *toxT* promoters relative to wild-type ToxR. This corresponded to a slight, but significant (p<0.05) increase in transcriptional activation of both of these promoters (Fig 2). Based on all of the wing mutants tested for DNA binding, ToxR activity at both the *toxT* and *ompU* promoters corresponded to the ability of the ToxR wing to bind these promoters.

### Some wing residues of ToxR affect TcpP interaction

Since previous studies have suggested ToxR and TcpP interaction plays a role in *toxT* activation [2, 26], we assessed the ability of several chromosomally-encoded ToxR mutants for their ability to interact with TcpP.

**Fig 6:**
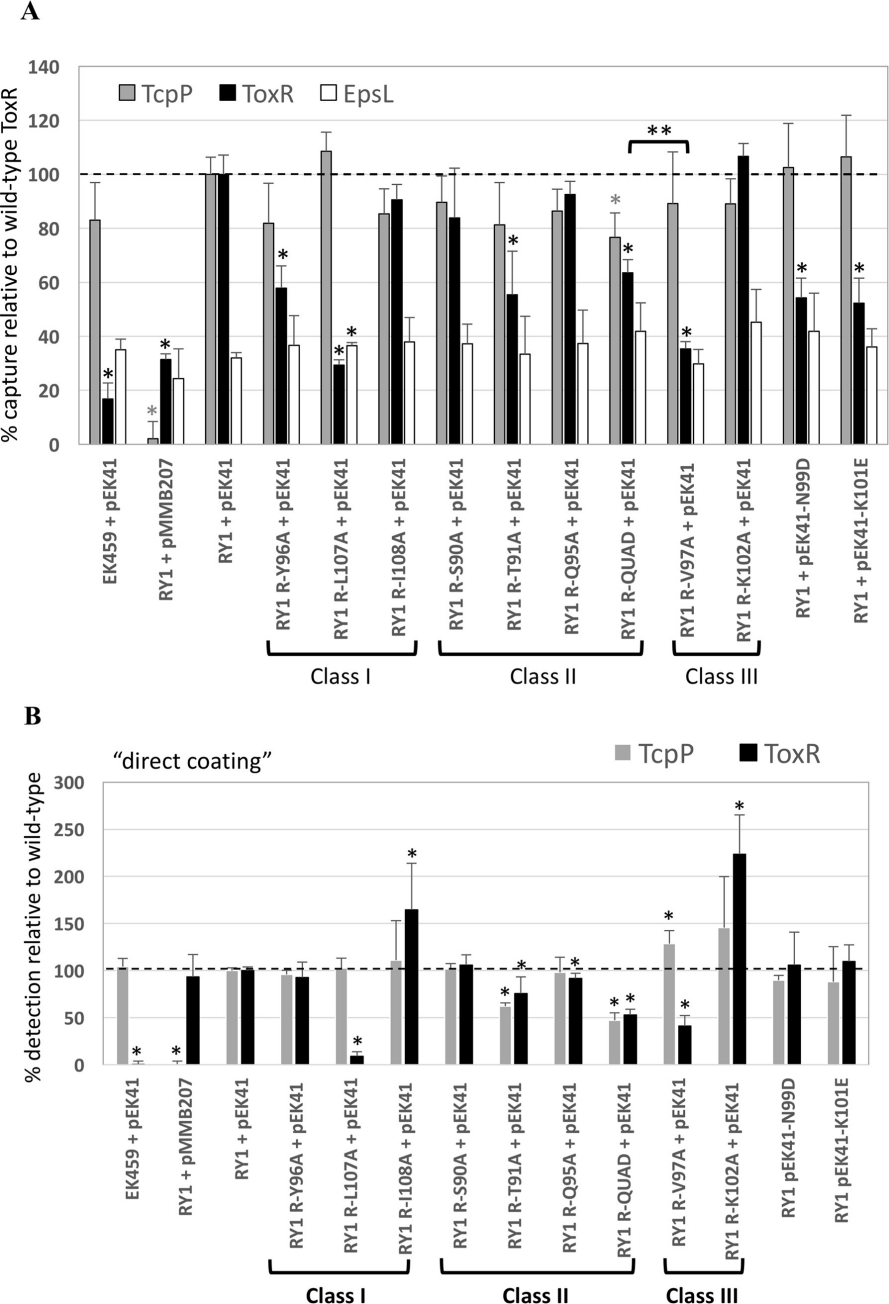
Mutations in most ToxR wing residues do not affect ToxR-TcpP interaction. Using membrane extracts expressing TcpP-HSV (from pEK41) and various chromosomally-expressed ToxR mutants, the ability of TcpP-HSV immobilized to a microtiter plate via anti-HSV antibodies to co-capture ToxR or the negative control inner membrane protein, EpsL was assessed. *V*. *cholerae* membranes were precipitated, resuspended at 2mg/ml protein and crosslinked with DSP prior to Triton X-100 solubilization of the membranes and ELISA capture of TcpP-HSV on mouse anti-HSV-coated 96-well plates. A) The levels of TcpP, ToxR and EspL were determined using specific rabbit anti-ToxR, rabbit anti-TcpP and rabbit anti-EpsL antibodies. Levels of each protein were normalized to the levels in a strain expressing wild-type TcpP-HSV and wild-type ToxR (set to 100%). The limit of detection for ToxR co-capture was about 17% based on a control capture assay with a TcpP-HSV^+^/ToxR^-^ strain (EK459 + pEK41). For EpsL, the co-capture raw data ELISA signal was normalized to the ToxR ELISA signal. With this normalization, the EpsL signal in RY1 + pEK41 was 32% (relative to ToxR) and levels of EpsL co-capture did not vary +/- TcpP, indicating a lack of EpsL binding by TcpP-HSV.* p<0.0035 relative to wild-type TcpP or ToxR using the Students’ T-test and a cut-off for significance at 0.05/13 according to the Bonferroni correction for multiple comparisons. EK459 = *V*. *cholerae* Δ*toxR*Δ*tcpP*, RY1 = Δ*tcpP*. B) Membrane extracts directly coated onto 96-well plates without anti-HSV antibodies were used to assess total levels of TcpP and ToxR in each extract. * p<0.05 relative to wild-type TcpP or ToxR. ** p<0.001 between ToxR-QUAD and ToxR-V97A. A dotted line was added at the 100% point on the Y-axis.

Two Class I mutants that expressed lower levels of *toxT* relative to *ompU* when expressed from a plasmid (Fig 2), ToxR-Y96A and ToxR-L107A, showed a 40% and 70% reduction in TcpP interaction (respectively) relative to wild-type ToxR using a ToxR co-capture assay where interacting membrane proteins are crosslinked with DSP, TcpP-HSV is immobilized onto plates using an anti-HSV mouse monoclonal antibody and the amount of ToxR co-captured is determined using a rabbit anti-ToxR polyclonal antibody (Fig 6, [2]). However, it should be noted, ToxR-L107A was barely detectable in whole cell extracts by direct coating of extracts onto a microtiter plate (Fig 6B). Thus, the fact that less ToxR-L107A was recruited is to be expected. However, it should be noted that the levels of both ToxR and TcpP in the ToxR-T91A membrane protein extract were also reduced relative to the wild-type ToxR extracts (Fig 6B and S2 Fig). This could explain the lower levels of ToxR co-capture by ToxR-T91A. On the other hand, ToxR-Y96A was expressed to similar levels as wild-type ToxR (Fig 6B and S2 Fig), but had a 40% defect in ToxR/TcpP interaction (Fig 6A), suggesting it has a bone fide defect in TcpP interaction.

One of three Class II mutants tested that showed no defect in *toxT* activation, ToxR-T91A (Figs 2–4), also had a 45% reduction in TcpP interaction (Fig 6A). But, ToxR-T91A also had reduced ToxR and TcpP levels in whole extracts (Fig 6B). Finally, one of the two Class III mutants tested that lacked DNA-binding activity (Fig 5) and the ability to activate either the *toxT* or *ompU* promoter (Figs 2–4), ToxR-V97A, was >60% defective for TcpP interaction. However, ToxR-V97A was also reduced in ToxR expression levels in whole cell extracts (Fig 6B). One should note that ToxR-QUAD showed a similar reduced level of ToxR in whole cell extracts (Fig 6B), yet maintained more TcpP interaction than ToxR-V97A (Fig 6A, p<0.001).

Two previously characterized mutants of TcpP (TcpP-N99D and TcpP-K102E) with reduced interaction with ToxR [2] served as negative controls and had ~50% reductions in ToxR interaction in the current assay (Fig 6). Low levels of EpsL detection, an inner membrane component of the Type II secretion apparatus that does not interact with TcpP were not affected by ToxR mutations or TcpP-HSV in this assay (Fig 6A, white bars).

## Discussion

Based on our findings, the wing of ToxR is required for DNA binding to both the *toxT* and *ompU* promoters. ToxR residues absolutely required for transcription activation of *toxT* and *ompU* (Fig 1, D89, V97, T99, K102, G104, and Y105) were also required for DNA binding (Fig 5 and [26]). Residues in the β-hairpin of the wing of ToxR are particularly critical for transcriptional activation and DNA binding (Figs 2–5). This is to be expected since this region of the wing is often inserted into the minor groove of the DNA [21, 25, 27]. Based on the modeled location of these residues (Fig 3), they may be involved in positioning of the wing relative to the rest of the w-HTH domain or directly interact with the DNA. K102 is predicted to protrude from the tip of the wing and likely interacts with nucleotides in the minor groove, similar to R219 in PhoB [21, 25]. ToxR-T99 and ToxR-Y105 correspond to PhoB-T217 and Y223 which interact with the backbone of DNA [21, 25]. ToxR-V97, ToxR-Y105, and ToxR-L107 may contribute to the hydrophobic core, since they correspond to residues that comprise the hydrophobic core in OmpR and PhoB [21, 24]. Furthermore, mutation of two of these residues (ToxR-V97A and ToxR-L107A) renders ToxR unstable (Fig 4), an anticipated phenotype of a mutant deficient in folding of the hydrophobic core. Mutation of a few residues near the hinge of the β-hairpin (ToxR-Y96, ToxR-L107 and ToxR-I108), where it joins the rest of the w-HTH domain, were preferentially required for *toxT* expression when expressed from a plasmid (Fig 2). However, these mutants lost the differential defect in *toxT* expression when expressed from the chromosomal *toxR* locus (Fig 4). It is likely that these residues are involved in positioning of the wing relative to the DNA-binding helix. I-TASSER and Chimera modeling of ToxR, based on other members of the w-HTH family [26], places many of these residues aimed towards the core of the protein indicating that they are likely involved in positioning of the wing.

D89, a residue in the loop leading from the DNA-binding helix (α3) to the β-hairpin of the wing was critical for DNA binding and activation of *toxT* and *ompU* (Figs 2, 4 and 5 and [26]). It is possible D89 contributes to the hydrophobic core of ToxR, but this is not likely since no residues in this loop have been shown to be part of the hydrophobic core in either OmpR or PhoB [21, 24]. We hypothesize D89 may be involved in positioning the wing relative to the DNA-binding helix.

Since mutation of residues C-terminal to amino acid 109 (ToxR-A109) had minimal effect on transcriptional activation of either promoter, it is likely that R110 marks the beginning of the variable linker region that tethers the w-HTH domain to the membrane [38].

Although some residues in the wing of ToxR were preferentially required for *toxT* activation, all of the residues tested lost this preferential requirement when expressed from the chromosome. Therefore, the preferential requirement of the wing for *toxT* activation is likely an effect of the overexpression system and not an actual increased dependence on the wing for *toxT* activation. Furthermore, the residues in the wing do not appear to preferentially be required for binding to one promoter or the other, since mutations leading to disruption of binding to the *toxT* promoter result in similar disruption of binding to the *ompU* promoter and the consensus sequences for ToxR binding to *toxT* and *ompU* are similar [4]. Additionally, a majority of the residues required for DNA binding are present in regions of the wing not likely to come into direct contact with the DNA, but instead are most likely involved in positioning of the wing such that it is able to insert into the minor groove. Together this shows that the role of the wing is to stabilize ToxR binding to promoter DNA.

Alanine substitutions in four ToxR residues were found to affect ToxR-TcpP interaction via a TcpP co-capture assay (Fig 6; ToxR-Y96A, ToxR-L107A, ToxR-T91A, and ToxR-V97A). We hypothesize general structural defects in some mutants may have resulted in disruption of the interaction with TcpP (V97A and L107A are both partially unstable and had some of the strongest defects in ToxR/TcpP interactions (Figs 4 and 6)). Alternatively, ToxR interacts with TcpP using some of the same wing residues required to bind DNA. In addition, Western blot analysis of protein extracts used in the capture assay and direct coating ELISAs to assess total protein content in these protein extracts showed three of four mutants defective for TcpP interaction had less ToxR available for TcpP interaction (Fig 6B and S2 Fig; ToxR-T91A, ToxR-V97A, and ToxR-L107A). The ToxR-T91A extract even had a reduced level of TcpP-HSV expression. This could account for the resulting decrease in ToxR/TcpP interaction for these mutants. ToxR-Y96A expressed similar levels of ToxR to wild-type ToxR and similar levels of TcpP-HSV, yet it still showed a 40% defect in TcpP interaction. ToxR-Y96A also had a preferential defect for *toxT* activation when expressed from a plasmid (Fig 2), but had no significant preferential defect in *toxT* activation when expressed from the chromosome (Fig 4). While these data argue against a direct role for the ToxR wing in TcpP interaction and TcpP recruitment to the *toxT* promoter, it is possible we have not yet identified a ToxR wing mutant allele that encodes a molecule fully capable of binding DNA, but defective for TcpP interaction.

**Fig 7:**
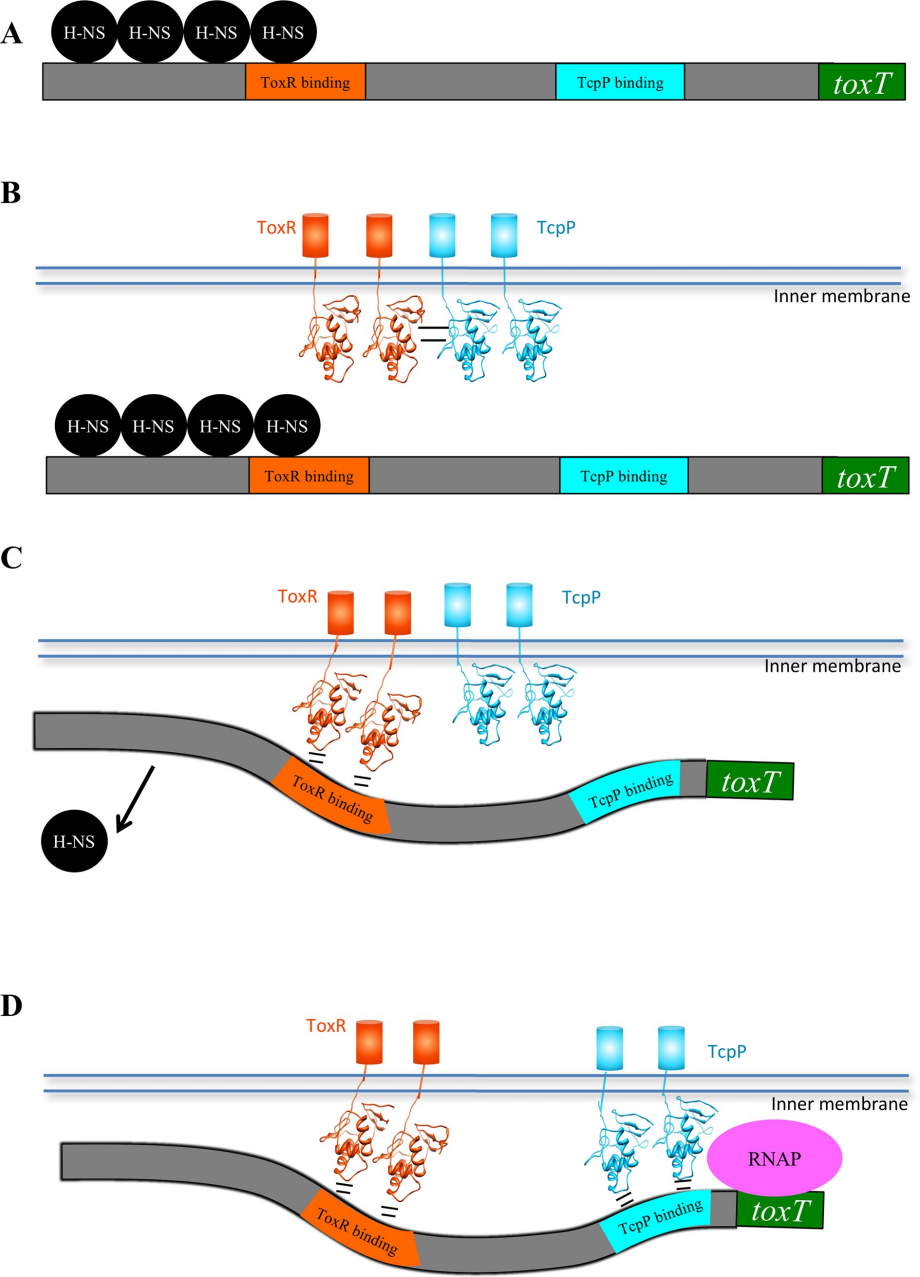
Model of ToxR mediated co-activation of the *toxT* promoter. A) Prior to activation, the *toxT* promoter is silenced by H-NS [35]. B) ToxR-TcpP interaction between the N-terminal β-sheet of ToxR and the wing of TcpP recruits TcpP to the *toxT* promoter. C) Upon ToxR binding to the *toxT* promoter, ToxR and TcpP no longer interact. ToxR binding to the promoter results in relieving H-NS repression, possibly bending the DNA to make the TcpP binding site more accessible [34], and recruiting the *toxT* promoter to the membrane [37]. D) This results in enhanced TcpP binding to the *toxT* promoter and transcriptional activation via TcpP interaction with RNA polymerase.

We propose that when ToxR binds the *toxT* promoter, TcpP is released to bind its neighboring TcpP-binding site on the *toxT* promoter. This “promoter delivery” model may allow ToxR to guide TcpP to its relatively weak TcpP-binding site (Fig 7C and 7D, [34]).

If ToxR were to maintain interaction with TcpP while bound to DNA, there are two likely ways in which ToxR and TcpP could interact based on the interaction of other w-HTH proteins: ToxR N-terminal β-sheet to TcpP N-terminal β-sheet or ToxR β-sheet to TcpP wing [21, 23, 28, 30, 31]. Previous studies have shown that the wing of TcpP is required for ToxR-TcpP interaction, since mutation of several residues of the wing of TcpP results in decreased ToxR interaction [2]. In this model, ToxR and TcpP would dimerize by interaction of the wing of TcpP with the β-sheet of ToxR (Fig 7B). If ToxR and TcpP interact in this orientation on the promoter it would place the wing of TcpP oriented upstream, away from the promoter, in the opposite orientation to other w-HTH proteins bound to their promoters [21, 25, 28]. Additionally, ToxR and TcpP bind to the *toxT* promoter three helical turns apart, making it difficult to envision how the w-HTH domains could interact when bound to the DNA [4], although ongoing studies indicate ToxR may in fact bind to these three intervening turns of the DNA helix (albeit with reduced affinity; ESK and Dr. Miquel Coll, unpublished observations). Finally, DNA binding by TcpP appears to be required for *toxT* activation [1] and the wing of TcpP is required for DNA binding [2, 34]. Thus, ToxR-TcpP interaction may enhance activation by recruiting TcpP to the *toxT* promoter along with ToxR, but this interaction may be disrupted upon ToxR DNA binding to allow TcpP to access its binding site downstream of ToxR on the *toxT* promoter (Fig 7). This would alleviate the need for ToxR and TcpP to interact while bound to DNA.

It has been previously shown that ToxR co-activation of *toxT* is due in part to alteration of the *toxT* promoter. ToxR relieves H-NS repression, recruits the promoter to the membrane, and exposes a DNAse I hypersensitivity site at the TcpP-binding site [34, 35, 37]. All of these functions are dependent on ToxR binding to the promoter, making the wing critical for ToxR co-activation of the *toxT* promoter. Based on this information we propose a model whereby ToxR-TcpP interaction recruits TcpP to the promoter. Upon ToxR binding to the *toxT* promoter (via the recognition helix and wing domain) the ToxR-TcpP interaction is disrupted. At this point H-NS repression is relieved due to displacement of H-NS by ToxR and TcpP is released in close proximity to its binding site on the *toxT* promoter. Given that at physiological concentrations TcpP is not as efficient at promoter binding as ToxR [34], the delivery of TcpP to the *toxT* promoter by ToxR may facilitate TcpP interactions with the *toxT* promoter (Fig 7). Single-molecule studies have also indicated that ToxR enhances the mobility of TcpP in the cell, facilitating its ability to reach the *toxT* promoter [36].

## Materials and methods

### Culture conditions

*V*. *cholerae* strains were routinely grown overnight in Vc LB (LB containing 5 g/L NaCl) at 37°C. Unless otherwise stated cultures were induced by dilution into Vc LB pH 6.5 and grown at 30°C. Cultures were grown in the presence of 100 μg/ml ampicillin, 25 μg/ml chloramphenicol, or 100 μg/ml streptomycin as needed.

### Construction of strains and plasmids

ToxR wing mutants were generated by PCR using complimentary mutagenic primers as described previously [4]. Chromosomal ToxR wing mutants were created using chromosomal recombination of the suicide plasmid pKAS32 and selected for by loss of streptomycin resistance as described previously [39]. A list of strains and plasmids used can be found in S1 Table and a list of primers used in this study can be found in S2 Table.

### Generation of an anti-ToxR antibody

A new anti-ToxR antibody was generated in two different rabbits by Covance using a His_6_-tagged ToxRcyt2 molecule containing the N-terminal 170 amino acids of ToxR including the winged-HTH domain and the linker domain prior to the transmembrane domain. Both animals gave a strong response to the antigen. This antibody was used to detect ToxR expressed from its chromosomal locus in ß-galactosidase assays (Fig 3) and capture assays (Fig 5).

### β-galactosidase assay for transcriptional activation by ToxR

*V*. *cholerae* strains containing chromosomal *ompU-lacZ* or *toxT-lacZ* reporters [2, 3] were diluted 1:30 and induced as described above. ToxR expression from pSK-ToxR-HA constructs in the presence of the repressing plasmid pREP4 was induced by addition of 100μM IPTG. For all transcriptional activation assays, activation was assayed after 4 hours by β-galactosidase assay as described previously [40]. The OD_600_ was determined by spectrophotometry and used to normalize cultures for subsequent Western blot analysis using antibodies against HA (Covance) or ToxR (generated against the N-terminal 170 amino acids of ToxR).

### Co-capture of ToxR with TcpP-HSV

Interactions between ToxR and TcpP were performed largely as previously described {Krukonis, 2003 #5067}{Morgan, 2011 #5620}, with some modifications. *V*. *cholerae* RY1 with each *toxR* allele of interest recombined at the normal *toxR* locus were transformed with plasmids expressing wild-type TcpP-HSV or the ToxR-interacting mutants TcpP-N99D or TcpP-K101E. Strains were diluted from an overnight culture 1:50 at 30°C and grown for 4–6 hrs at 30°C in 500ml LB containing 100μg/ml streptomycin, 25μg/ml chloramphenicol and 100μM IPTG. Membranes containing ToxR mutant proteins and/or TcpP-HSV proteins were prepared [41] and dialyzed into HEPES-buffered saline (HBS, 20mM HEPES pH = 7.0, 150 mM NaCl). Membranes were then treated for 30 minutes with 1 μl DNase I (New England Biolabs) in the presence of 2.5mM MgCl_2_ and 0.5 mM CaCl_2_. 2 mg/ml membrane proteins were then crosslinked using a 15-fold molar excess of DSP (Pierce) for 30 minutes at room temp, blocked with 50mM Tris pH = 7.4 and then solubilized in 1% Triton X-100 (Bio-Rad). The molarity of dialyzed membrane preparations was estimated by measuring the protein concentration and assuming a 50kD average protein size in the total membrane extract. Samples were then sonicated on ice (3 x 5 seconds) and 50μl membrane extracts were added to microtiter plates coated with mouse an anti-HSV antibody (Novagen, coated at 1:500 dilution in PBS). Membrane extract binding to the plate occurred overnight at 4°C. After washing five times with PBS, wells were incubated with 50μl a 1:250 dilution of rabbit anti-TcpP antibody, 1:1000 dilution rabbit anti-ToxR antibody (a kind gift from Dr. Victor DiRita) or 1:10,000 dilution rabbit anti-EpsL antibody (a kind gift from Dr. Maria Sandkvist). Primary antibody incubation proceeded overnight at 4°C. After five washes with PBS, wells were then incubated with a 1:3000 dilution of goat anti-rabbit-AP conjugated secondary antibody (Zymed) and binding was detected by addition of 100μl of the colorimetric substrate PNPP (Sigma) at 4 mg/ml following sequential washing with PBS (four times), ansd Tris-buffered saline (100mM Tris pH = 8.0, 150mM NaCl, one wash). Plates were read at ABS_405_. Relative levels of each ToxR mutant protein in the membrane extract were assessed by directly-coating samples on 96-well ELISA plates and using an anti-ToxR polyclonal antibody at a 1:1000 dilution. Relative levels of TcpP-HSV in each strain were assessed using the anti-TcpP monoclonal antibody at a 1:250 dilution. The amount of TcpP-HSV or ToxR captured is presented as % of wild-type. Samples diluted on the same day to the same relative concentrations used in the capture assay were boiled in SDS-sample buffer and run in a 12% SDS-PAGE gel for Western blot analysis of protein levels in the extracts. Statistical analysis was performed using the students’ t-test followed by a Bonferroni correction for multiple comparisons (0.05/11 comparisons = 0.0045), with six measurements from two different days relative to the strain expressing wild-type ToxR and wild-type TcpP-HSV. Levels of EpsL were expressed based on the raw ABS_405_ values and normalized to the raw ABS_405_ values of ToxR developed for the sample length of time (45 minute reaction) in the RY1 + pEK41 sample.

### DNA mobility shift assay

DNA binding assays were performed as described previously [34]. Membranes were isolated from Δ*toxR*Δ*tcpP V*. *cholerae* strains (EK459) with pSK-*toxR-HA* alleles which had been induced for 6 hours in 1mM IPTG as described previously [41]. Membrane concentrations ranging from 0.19 to 2 mg/ml were incubated with either the *ompU* promoter probe (extending from -211 to +22 relative to the transcriptional start site), the *toxT* promoter probe (extending from -172 to +45 relative to the transcriptional start site), or the negative control probe (*toxT* promoter extending from-46 to +45 relative to the transcriptional start site). 3000 cpm of probe labeled with ^32^P-dATP was used in each reaction. Western blotting with anti-HA (Covance) antibody was used to monitor ToxR protein levels.

### Modeling of ToxR wing residues

ToxR bound to DNA was modeled using the I-TASSER modeling program (http://zhanglab.ccmb.med.umich.edu/I-TASSER/) to determine ToxR structure based on the crystal structure of other w-HTH family members [26]. Binding of ToxR to DNA was modeled using the NMR structure of PhoB bound to DNA [25]. ToxR wing residues were modeled using the program Chimera based on the threaded structure of ToxR as determined previously by I-TASSER [26].

## Supporting information

S1 Fig Class I and III mutants are defective for binding both the *ompU* and *toxT* promoters. DNA binding by ToxR wing mutants was assayed by EMSA using *V*. *cholerae* membranes containing ToxR wing mutants, but lacking TcpP (See Fig 4). Band intensity of the shifted and unshifted fractions was measured by Image J. The percent shifted was defined as the unshifted fraction divided by the sum of the shifted and unshifted fractions. For *ompU* the intermediate ToxR-independent shifted fraction which shifts in the absence of ToxR was counted as unshifted. Percent shifted is plotted against the membrane protein concentration used as determined by Bradford Assay.
(PDF)

S2 Fig Western blot analysis of TcpP, ToxR, and EpsL levels loaded from the same extracts used in the capture assay (Fig 5). Levels reflect the results of the direct coating ELISA (Fig 5B) and show similar levels of EpsL in all extracts.
(PDF)

S1 Table Strains and Plasmids. (PDF)

S2 Table Primers used in this study. (PDF)
