# Supplementary material for: The wing of the ToxR winged helix-turn-helix domain is required for DNA binding and activation of toxT and ompU
Source: PLoS One. 2019 Sep 9;14(9):e0221936. doi: 10.1371/journal.pone.0221936 (PMC6733452; doi:10.1371/journal.pone.0221936)
Supplement: S1 Fig — DNA binding by ToxR wing mutants was assayed by EMSA using V. cholerae membranes containing ToxR wing mutants, but lacking TcpP (See Fig 4). Band intensity of the shifted and unshifted fractions was measured by Image J. The percent shifted was defined as the unshifted fraction divided by the sum of the shifted and unshifted fractions. For ompU the intermediate ToxR-independent shifted fraction which shifts in the absence of ToxR was counted as unshifted. Percent shifted is plotted against the membrane protein concentration used as determined by Bradford Assay. (PDF) [file pone.0221936.s001.pdf]

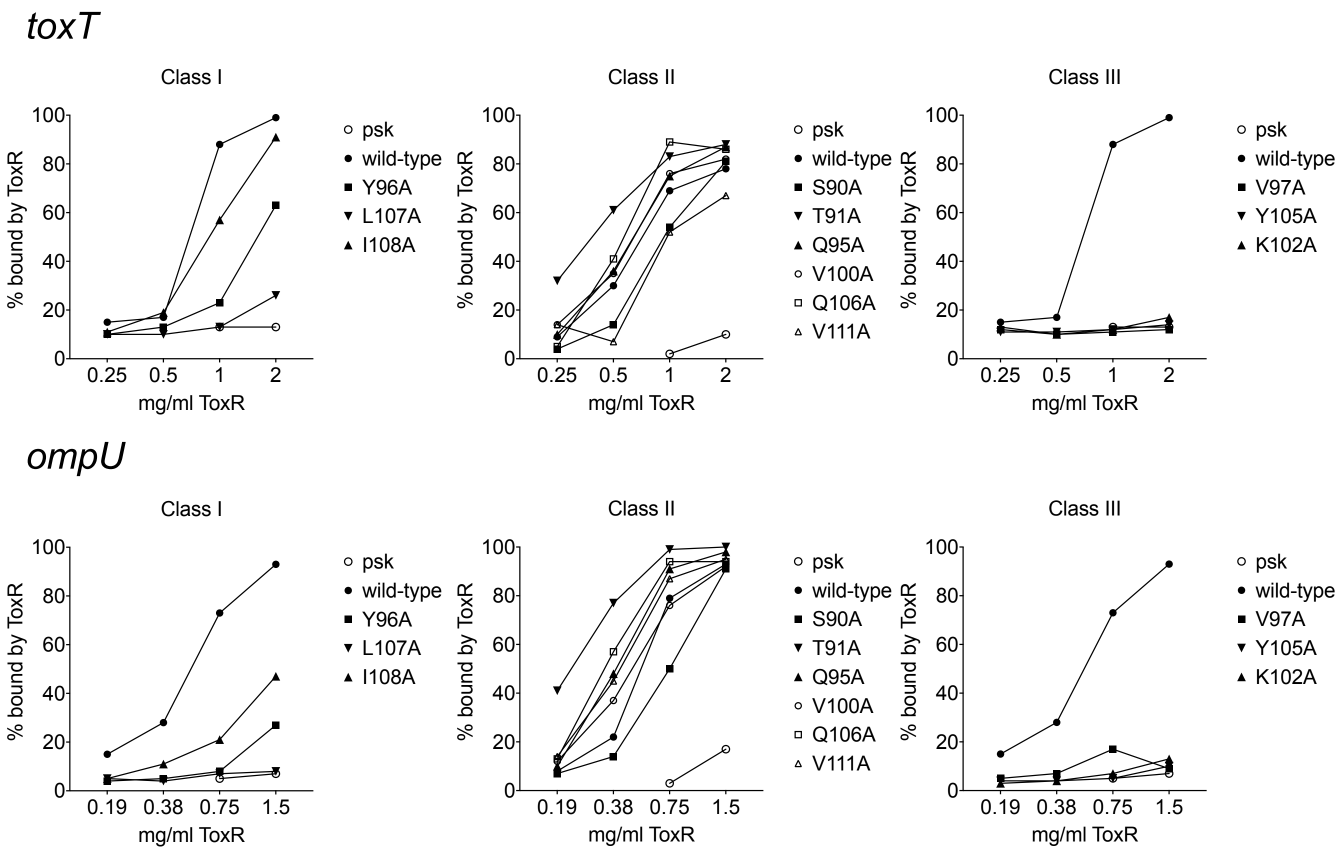

S1 Figure: **Class I and III mutants are defective for binding both the *ompU* and *toxT* promoters.** DNA binding by ToxR wing mutants was assayed by EMSA using *V. cholerae* membranes containing ToxR wing mutants, but lacking TcpP (See Fig 4). Band intensity of the shifted and unshifted fractions was measured by Image J. The percent shifted was defined as the unshifted fraction divided by the sum of the shifted and unshifted fractions. For *ompU* the intermediate ToxR-independent shifted fraction which shifts in the absence of ToxR was counted as unshifted. Percent shifted is plotted against the membrane protein concentration used as determined by Bradford Assay.
