# Supplementary material for: The wing of the ToxR winged helix-turn-helix domain is required for DNA binding and activation of toxT and ompU
Source: PLoS One. 2019 Sep 9;14(9):e0221936. doi: 10.1371/journal.pone.0221936 (PMC6733452; doi:10.1371/journal.pone.0221936)
Supplement: S2 Fig — Levels reflect the results of the direct coating ELISA (Fig 5B) and show similar levels of EpsL in all extracts. (PDF) [file pone.0221936.s002.pdf]

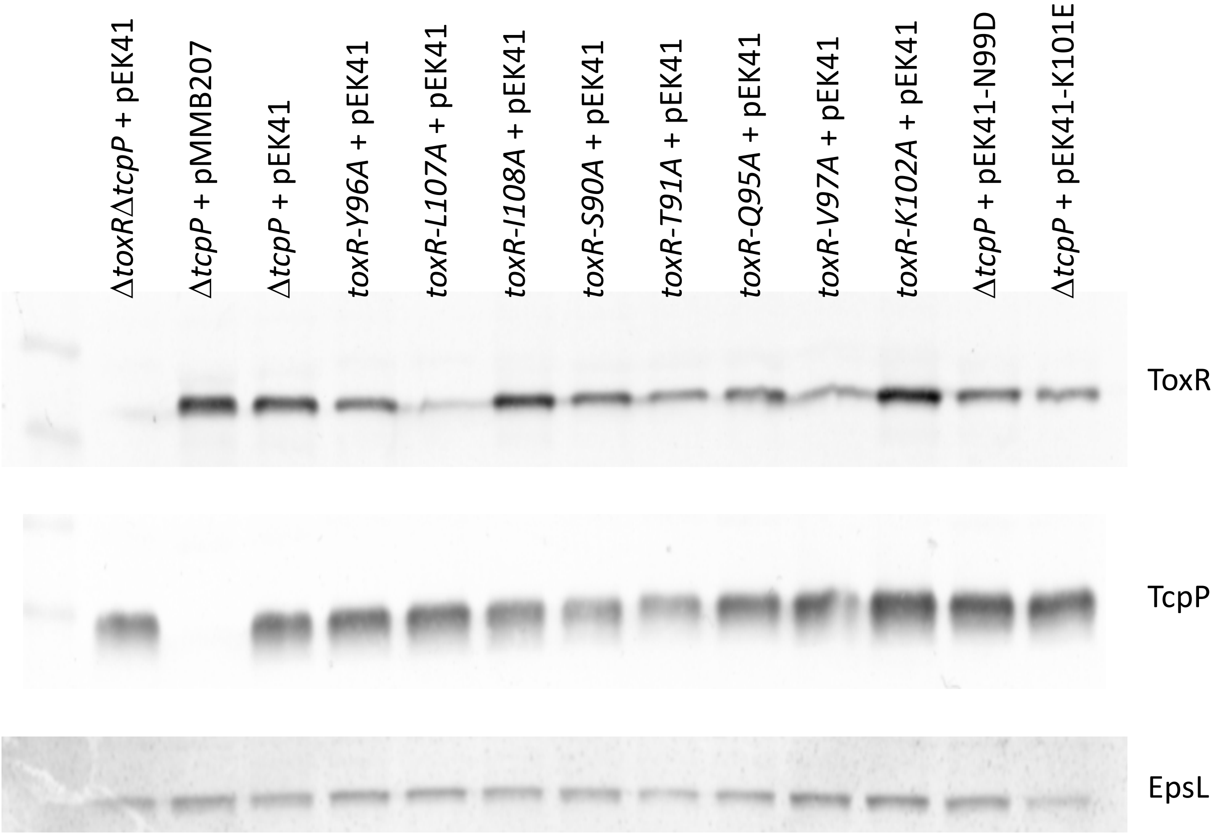

S2 Figure: Western blot analysis of TcpP, ToxR, and EpsL levels loaded from the same extracts used in the capture assay (Fig 5). Levels reflect the results of the direct coating ELISA (Fig 5B) and show similar levels of EpsL in all extracts.
