# Supplementary material for: The wing of the ToxR winged helix-turn-helix domain is required for DNA binding and activation of toxT and ompU
Source: PLoS One. 2019 Sep 9;14(9):e0221936. doi: 10.1371/journal.pone.0221936 (PMC6733452; doi:10.1371/journal.pone.0221936)
Supplement: S1 Table — (PDF) [file pone.0221936.s003.pdf]

S1 Table: Strains and Plasmids

***Vibrio cholerae* strains**	
0395 Δ*toxR toxT-lacZ* (EK1072)	(1)
0395 Δ*toxR ompU-lacZ* (EK410)	(2)
0395 *ompU-lacZ* (EK383)	(2)
0395 *ompU-lacZ toxR*-Y96A	This study
0395 *ompU-lacZ toxR*-L107A	This study
0395 *ompU-lacZ toxR*-I108A	This study
0395 *ompU-lacZ toxR*-S90A	This study
0395 *ompU-lacZ toxR*-T91A	This study
0395 *ompU-lacZ toxR*-Q95A	This study
0395 *ompU-lacZ toxR*-V100A	This study
0395 *ompU-lacZ toxR*-S90A/T91A/Q95A/V100A (quad)	This study
0395 *ompU-lacZ toxR*-V97A	This study
0395 *ompU-lacZ toxR*-K102A	This study
0395 *toxT-lacZ* (EK733)	(3)
0395 *toxT-lacZ toxR*-Y96A	This study
0395 *toxT-lacZ toxR*-L107A	This study
0395 *toxT-lacZ toxR*-I108A	This study
0395 *toxT-lacZ toxR*-S90A	This study
0395 *toxT-lacZ toxR*-T91A	This study
0395 *toxT-lacZ toxR*-Q95A	This study
0395 *toxT-lacZ toxR*-V100A	This study
0395 *toxT-lacZ toxR*-S90A/T91A/Q95A/V100A (quad)	This study
0395 *toxT-lacZ toxR*-V97A	This study
0395 *toxT-lacZ toxR*-K102A	This study
0395 Δ*tcpP* Δ*toxR* (EK459)	(4)
0395 Δ*tcpP* (RY1)	(5)
0395 Δ*tcpP toxR*-T91A	This study
0395 Δ*tcpP toxR*-L107A	This study
0395 Δ*tcpP toxR*-I108A	This study
0395 Δ*tcpP toxR*-S90A	This study
0395 Δ*tcpP toxR*-S90A/T91A/Q95A/V100A (quad)	This study
0395 Δ*tcpP toxR*-V97A	This study
0395 Δ*tcpP toxR*-K102A	This study
0395 Δ*tcpP toxR*-Q95A	This study
0395 Δ*tcpP toxR*-Y96A	This study
***E. coli* strains**	
DH5α	Lab strain
DHM1	(6)
BTH101	(7)

**Plasmids**	
pBluescriptSK(-)	Laboratory collection
pBluescriptSK(-)-*toxR-HA*	(1)
pMMB207	(8)
pMMB207-*tcpP-HSV*	(9)
pUT18c	(7)
pUT18c-ToxRS	(1)
pKT25	(7)
pKT25-TcpPH	(1)

References

1. Morgan SJ, Felek S, Gadwal S, Koropatkin NM, Perry JW, Bryson AB, Krukonis ES. 2011. The two faces of ToxR: activator of ompU, co-regulator of *toxT* in *Vibrio cholerae*. Mol Microbiol 81:113-128.
2. Crawford JA, Krukonis ES, DiRita VJ. 2003. Membrane localization of the ToxR winged-helix domain is required for TcpP-mediated virulence gene activation in *Vibrio cholerae*. Mol Microbiol 47:1459-73.
3. Häse CC, Mekalanos JJ. 1998. TcpP protein is a positive regulator of virulence gene expression in *Vibrio cholerae*. Proc Natl Acad Sci USA 95:730-734.
4. Krukonis ES, Yu RR, Dirita VJ. 2000. The *Vibrio cholerae* ToxR/TcpP/ToxT virulence cascade: distinct roles for two membrane-localized transcriptional activators on a single promoter. Mol Microbiol 38:67-84.
5. Yu RR, DiRita VJ. 1999. Analysis of an autoregulatory loop controlling ToxT, cholera toxin, and toxin-coregulated pilus production in *Vibrio cholerae*. J Bacteriol 181:2584-92.
6. Karimova G, Dautin N, Ladant D. 2005. Interaction network among *Escherichia coli* membrane proteins involved in cell division as revealed by bacterial two-hybrid analysis. J Bacteriol 187:2233-43.
7. Karimova G, Ullmann A, Ladant D. 2001. Protein-protein interaction between *Bacillus stearothermophilus* tyrosyl-tRNA synthetase subdomains revealed by a bacterial two-hybrid system. J Mol Microbiol Biotechnol 3:73-82.
8. Morales VM, Backman A, Bagdasarian M. 1991. A series of wide-host-range low-copy-number vectors that allow direct screening for recombinants. Gene 97:39-47.
9. Krukonis ES, DiRita VJ. 2003. DNA binding and ToxR responsiveness by the wing domain of TcpP, an activator of virulence gene expression in *Vibrio cholerae*. Mol Cell 12:157-65.
