# Supplementary material for: The wing of the ToxR winged helix-turn-helix domain is required for DNA binding and activation of toxT and ompU
Source: PLoS One. 2019 Sep 9;14(9):e0221936. doi: 10.1371/journal.pone.0221936 (PMC6733452; doi:10.1371/journal.pone.0221936)
Supplement: S2 Table — (PDF) [file pone.0221936.s004.pdf]

S2 Table. Primers used in this study

Name	Sequence
toxR D89A top	CGCAAAATGCTCAAAGCTTCGACAAAGTCCC
toxR D89A bottom	GGGACTTTGTCGAAGCTTTGAGCATTTTGCG
ToxR S90A top	CAAAATGCTCAAAGATGCGACAAAGTCCCCAC
ToxR S90A bottom	GTGGGGACTTTGTCGCATCTTTGAGCATTTTG
ToxR T91A top	ATGCTCAAAGATTCGGCAAAGTCCCCACAATAC
ToxR T91A bottom	ATTGTGGGGACTTTGCCGAATCTTTGAGCATTTTG
toxR K92A top	CTCAAAGATTCGACAGCGTCCCCACAATACGTC
toxR K92A bottom	GACGTATTGTGGGGACGCTGTCGAATCTTTGAG
toxR S93A top	CAAAGATTCGACAAAGGCCCCACAATACGTCAAAAC
toxR S93A bottom	GTTTTGACGTATTGTGGGGCCTTTGTCGAATCTTTG
toxR P94A top	GATTCGACAAAGTCCGCACAATACGTCAAAAC
toxR P94A bottom	GTTTTGACGTATTGTGCGGACTTTGTCGAATC
ToxR Q95A top	TCGACAAAGTCCCCAGCATACGTCAAAACGGTTC
ToxR Q95A bottom	GAACCGTTTTGACGTATGCTGGGGACTTTGTCGAATC
ToxR Y96A top	GACAAAGTCCCCACAAGCCGTCAAAACGGTTC
ToxR Y96A bottom	GAACCGTTTTGACGGCTTGTGGGGACTTTGTC
ToxR V97A top	CAAGTCCCCACAATACGCCAAAACGGTTCCGAAG
ToxR V97A bottom	CTTCGGAACCGTTTTGGCGTATTGTGGGGACTTG
toxR K98A top	GTCCCCACAATACGTCGCAACGGTTCCGAAGCG
toxR K98A bottom	CGCTTCGGAACCGTTGCGACGTATTGTGGGGAC
toxR T99A top	CACAATACGTCAAAGCGGTTCCGAAGCGCGG
toxR T99A bottom	CCGCGCTTCGGAACCCGTTTGACGTATTGTG
ToxR V100A top	CAATACGTCAAAACGGCTCCGAAGCGCGGTTAC
ToxR V100A bottom	GTAACCGCGCTTCGGAGCCGTTTTGACGTATTG
toxR P101A top	CAATACGTCAAAACGGTTGCGAAGCGCGGTTACC
toxR P101A bottom	GGTAACCGCGCTTCGCAACCGTTTTGACGTATTG
toxR K102A top	GTCAAAACGGTTCCGGCGCGCGGTTACCAATTG
toxR K102A bottom	CAATTGGTAACCGCGCGCCGGAACCGTTTTGAC
toxR R103A top	CAAAACGGTTCCGAAGGCCGGTTACCAATTGATC
toxR R103A bottom	GATCAATTGGTAACCGGCCTTCGGAACCGTTTTG
toxR G104A top	CGGTTCCGAAGCGCGCTTACCAATTGATCGC
toxR G104A bottom	GCGATCAATTGGTAAGCGCGCTTCGGAACCG
ToxR Y105A top	GTTCCGAAGCGCGGTGCCCAATTGATCGCCCGAG
ToxR Y105A bottom	CTCGGGCGATCAATTGGGCACCGCGCTTCGGAAC
ToxR Q106A top	CCGAAGCGCGGTTACGCATTGATCGCCCGAGTG
ToxR Q106A bottom	CACTCGGGCGATCAATGCGTAACCGCGCTTCGG
ToxR L107A top	GAAGCGCGGTTACCAAGCGATCGCCCGAGTGGAAAC
ToxR L107A bottom	GTTTCCACTCGGGCGATCGCTTGGTAACCGCGCTTC
ToxR I108A top	CGCGGTTACCAATTGGCCGCCCGAGTGGAAAC

ToxR I108A bottom	GTTTCCACTCGGGCGGCCAATTGGTAACCGCG
ToxR R110A top	GTTACCAATTGATCGCCGCAGTGGAAACGGTTGAAG
ToxR R110A bottom	CTTCAACCGTTTCCACTGCGGCGATCAATTGGTAAC
ToxR V111A top	CAATTGATCGCCCGAGCGGAAACGGTTGAAGAAG
ToxR V111A bottom	CTTCTTCAACCGTTTCCGCTCGGGCGATCAATTG
ToxR E112A top	GATCGCCCGAGTGGCAACGGTTGAAGAAGAG
ToxR E112A bottom	CTCTTCTTCAACCGTTGCCACTCGGGCGATC
ToxR T113A top	GATCGCCCGAGTGGAAGCGGTTGAAGAAGAGATG
ToxR T113A bottom	CATCTCTTCTTCAACCGCTTCCACTCGGGCGATC
ToxR V114A top	GCCCGAGTGGAAACGGCTGAAGAAGAGATGGCTC
ToxR V114A bottom	GAGCCATCTCTTCTTCAGCCGTTTCCACTCGGGC
ToxR V111A top	CAATTGATCGCCCGAGCGGAAACGGTTGAAGAAG
ToxR V111A bottom	CTTCTTCAACCGTTTCCGCTCGGGCGATCAATTG
ToxR E112A top	GATCGCCCGAGTGGCAACGGTTGAAGAAGAG
ToxR E112A bottom	CTCTTCTTCAACCGTTGCCACTCGGGCGATC
ToxR T113A top	GATCGCCCGAGTGGAAGCGGTTGAAGAAGAGATG
ToxR T113A bottom	CATCTCTTCTTCAACCGCTTCCACTCGGGCGATC
ToxR V114A top	GCCCGAGTGGAAACGGCTGAAGAAGAGATGGCTC
ToxR V114A bottom	GAGCCATCTCTTCTTCAGCCGTTTCCACTCGGGC
